# Timing of Adjuvant Chemotherapy and Survival in Colorectal, Gastric, and Pancreatic Cancer. A Systematic Review and Meta-Analysis

**DOI:** 10.3390/cancers11040550

**Published:** 2019-04-17

**Authors:** Fausto Petrelli, Alberto Zaniboni, Antonio Ghidini, Michele Ghidini, Luca Turati, Claudio Pizzo, Margherita Ratti, Michela Libertini, Gianluca Tomasello

**Affiliations:** 1 Oncology Unit, ASST Bergamo Ovest, 24047 Treviglio, Italy; 2 Oncology Unit, Fondazione Poliambulanza, 25124 Brescia, Italy; azaniboni@alice.it (A.Z.); michela.libertini@poliambulanza.it (M.L.); 3 Oncology Unit, Casa di Cura Igea, 20129 Milano, Italy; antonioghidini@hotmail.com; 4 Oncology Unit, ASST Cremona, 26100 Cremona, Italy; micheleghidini@outlook.com (M.G.); claupizz1987@gmail.com (C.P.); margherita.ratti@studenti.unipr.it (M.R.); gianluca.tomasello@gmail.com (G.T.); 5 Surgical Oncology Unit, ASST of Bergamo, 24100 Bergamo Ovest, Italy; luca_turati@ospedale.treviglio.bg.it

**Keywords:** colorectal cancer, gastric cancer, pancreatic cancer, adjuvant chemotherapy, timing, meta-analysis

## Abstract

(1) Background: The optimal timing of adjuvant chemotherapy (CT) in gastrointestinal malignancies is still a matter of debate. For colorectal cancer, it is recommended to start post-operative treatment within eight weeks. The objective of this study was to assess the clinical effects of starting adjuvant CT within or after 6–8 weeks post-surgery in colorectal, gastric, and pancreatic cancer. (2) Methods: MEDLINE, EMBASE, and the Cochrane Library were searched in December 2018. Publications comparing the outcomes of patients treated with adjuvant CT administered before (early) or after (delayed) 6–8 weeks post-surgery for colorectal, gastric, and pancreatic cancer were identified. The primary endpoint was overall survival (OS). (3) Results: Out of 8752 publications identified, 34 comparative studies assessing a total of 141,853 patients were included. Meta-analysis indicated a statistically significant increased risk of death with delayed CT (>6–8 weeks post-surgery) in colorectal cancer (hazard ratio (HR) = 1.27, 95% confidence interval (CI) 1.21–1.33; *p* <0.001). Similarly, for gastric cancer, delaying adjuvant CT was associated with inferior overall survival (HR = 1.2, 95% CI 1.04–1.38; *p* = 0.01). Conversely, the benefit of earlier CT was not evident in pancreatic cancer (HR = 1, 95% CI 1–1.01; *p* = 0.37). Conclusions: Starting adjuvant CT within 6–8 weeks post-surgery is associated with a significant survival benefit for colorectal and gastric cancer, but not for pancreatic cancer.

## 1. Introduction

Surgical resection is the mainstay of treatment for most solid malignancies diagnosed at the localized stage. Unfortunately, disease recurrence is frequently encountered and mainly depends on the presence of clinically occult micrometastases at the time of surgery. Post-operative chemotherapy (CT) aims to eradicate these, thereby decreasing the possibility of recurrence.

The benefits of adjuvant CT have been clearly demonstrated in major gastrointestinal malignancies. Specifically, adding oxaliplatin to fluoropyrimidines has been associated with a significant survival gain in stage III radically resected colorectal cancer patients [1]. In gastric cancer, one of the largest meta-analyses concluded that adjuvant systemic therapy was associated with a 15% reduction in risk of death compared with surgery alone [2]. Finally, a very recent randomized phase III study compared an intensified triplet combination CT regimen (i.e., modified 5-fluorouracil, irinotecan, and oxaliplatin (FOLFIRINOX)) with the standard of care (i.e. Gemcitabine) in patients with resected pancreatic cancer. Although more toxic, FOLFIRINOX was shown to significantly improve both disease-free and overall survival [3].

The optimal time to initiate adjuvant CT is yet to be established and postoperative treatment is typically started once the patient has recovered from surgery. Major adjuvant randomized studies recommend initiation of CT within six to eight weeks after resection, and this has become an accepted approach. However, there is a lack of prospective trials that specifically evaluate whether starting the administration of adjuvant therapy after eight weeks compromises outcomes. To answer this important clinical question, we performed a systematic review of all available studies and a meta-analysis in gastrointestinal and pancreatic cancer settings.

## 2. Results

Our systematic literature search retrieved 8752 studies, 34 of which matched our inclusion criteria. These 34 studies corresponded to the post-hoc analyses of randomized controlled trials and cohorts and to retrospective studies. They included a total of 141,853 patients: 134,701 in the colon cancer cohort, as shown in Table 1 [4,5,6,7,8,9,10,11,12,13,14,15,16,17,18,19,20,21,22,23,24,25]; 5121 in the gastric cancer cohort, as shown in Table 2 [26,27,28,29,30,31]; and 2031 in the pancreatic cancer cohort, as shown in Table 3 [32,33,34,35,36,37]. Among these studies, 22 were used for the colorectal cancer analysis and six were used for pancreatic and gastric cancer each. Table 1, Table 2 and Table 3 show the main characteristics of the studies and the cut-offs of the timing of adjuvant chemotherapy evaluated in each study. When the multivariate analysis was performed, the confounding factors typical of each study were considered (i.e. tumor extension, stage, nodal status, age, and sex).

All studies were retrospective, except for three randomized phase 3 trials and one prospective study. All articles were fully published between 2005 and 2017. In *n* = 25 studies, the comparison was made between more than versus (vs) less than eight weeks, in *n* = 5 studies between more vs less than six weeks, in *n* = 3 studies between 6.5 vs < 6.5 weeks, and in *n* = 1 study between 5–6 vs less than 5–6 weeks. 

### 2.1. Effect of Delaying CT on Survival in Colorectal Cancer

**Figure 1:**
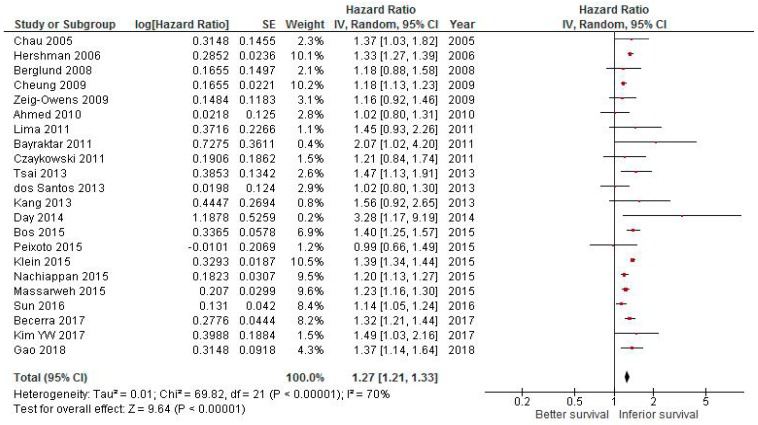
Forest plot of association between delay of adjuvant chemotherapy beyond 6–8 weeks and survival in colorectal cancer.

Among colorectal cancer studies, the combined hazard ratio (HR) for delayed vs earlier adjuvant CT was 1.27 (95% confidence interval (CI) 1.21–1.33; *p* <0.001; Figure 1).

As heterogeneity was found (*I*^2^ = 70%, *p* < 0.001), a random effect model was used. After removing the four studies with the largest weight sequentially, the HR ranged from 1.25 to 1.28 and remained significant in all cases. All studies except one reported the results as multivariate analyses.

### 2.2. Effect of Delaying CT on Survival in Gastric Cancer

**Figure 2:**
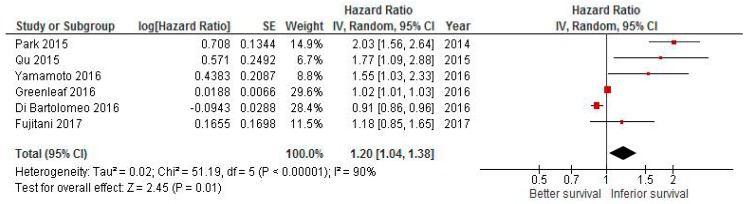
Forest plot of association between delay of adjuvant chemotherapy beyond 6–8 weeks and survival in gastric cancer.

By pooling the results of the six gastric cancer studies, the combined HR for delayed vs earlier adjuvant CT was 1.2 (95% CI 1.04–1.38; *p* = 0.01; Figure 2).

Again, there was evidence of heterogeneity (*I*^2^ = 90%, *p* < 0.001), therefore, a random effect model was used. After removing the study with the largest weight [28], the HR was 1.41 (95% CI 0.94–2.1, *p* = 0.09). All studies reported the results as multivariate analyses.

### 2.3. Effect of Delaying CT on Survival in Pancreatic Cancer

**Figure 3:**
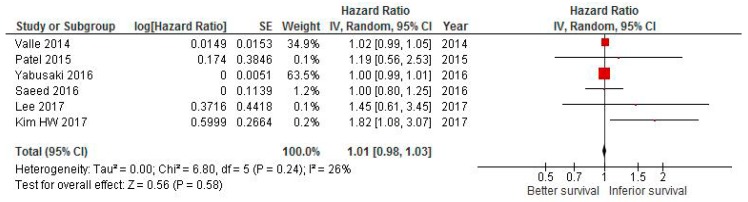
Forest plot of association between delay of adjuvant chemotherapy beyond 6–8 weeks and survival in pancreatic cancer.

The pooled HR attained from six studies in pancreatic cancer was 1 (95% CI 1–1.01, *p* = 0.37; Figure 3).

There was no evidence of heterogeneity (*I*^2^ = 20%, *p* <0.001), so a fixed effect model was used. Removing the largest study (Reference [37]) did not change the final result. 

### 2.4. Publication Bias

**Figure 4:**
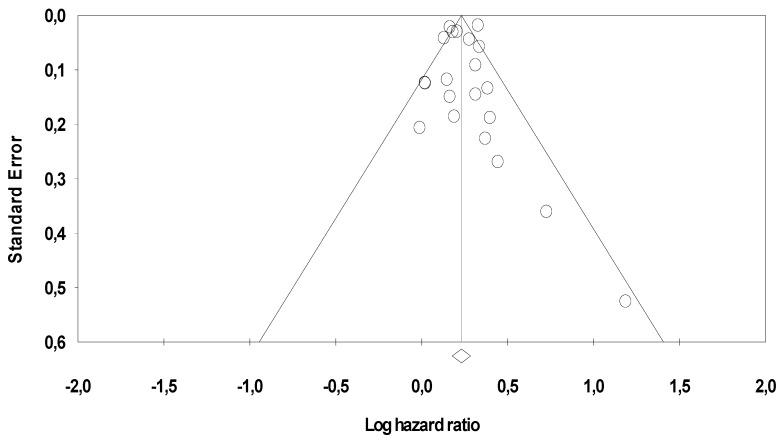
Funnel plot of the relationship between the log hazard ratio (HR) and standard error of the log HR for overall survival (OS) in colorectal cancer studies.

The funnel plot for the degree of asymmetry of the individual study results around the combined HR for overall survival (OS) in colorectal cancer studies is shown in Figure 4. 

The degree of asymmetry was not statistically significant according to the Egger method (*p* = 0.47). We used the trim and fill approach to adjust our estimate of effect size for potential asymmetry. The imputed estimate (HR =1.25; 95% CI, 1.18–1.32) was similar to that in the main analysis, indicating that the results are unlikely to be explained by publication bias.

## 3. Discussion

Most of the currently available evidence on the optimal timing of adjuvant CT is retrospective and derived from breast and colorectal cancer studies. For example, early initiation of adjuvant CT (within 20 days post-surgery) was shown to be associated with a significant improvement in disease-free survival in estrogen-receptor-negative premenopausal breast cancer patients [38]. Similarly, in stage III colorectal cancer, a recent study including 72,057 patients concluded that the maximum survival benefit of adjuvant CT was obtained when treatment was started within six to eight weeks [39]. Two previously published meta-analyses have also shown that delays beyond two months may compromise the effectiveness of CT [40,41].

In some cases, CT delays are caused by post-surgical complications and a retrospective cohort study of stage III colon cancer patients reported that 30–38% were surgeon-specific delays, while the vast majority were caused by medical oncologists and hospital-specific practices [6]. In a retrospective series of patients with stage I to III invasive breast cancer, it was mainly sociodemographic determinants that caused CT initiation delays of 91 or more days [42]. Interestingly, in cases of totally resected non-small-cell lung cancer, patients still benefited from delayed adjuvant CT when therapy was started up to four months after surgery [43]. It is not clear, however, whether there is any time point beyond which the benefits of adjuvant CT are lost for gastric and pancreatic cancers.

The results of this meta-analysis, which evaluated 34 studies and 141,853 patients, indicated that delaying the initiation of adjuvant CT beyond eight weeks post-surgery was associated with a 27% and 20% increased risk of death for colorectal and gastric cancer, respectively. For pancreatic cancer, no statistically significant difference was found for patients starting earlier compared with patients receiving post-operative treatment after two months. Noteworthy, the detrimental effect of delayed starting of adjuvant CT was independent by other main clinicopathological risk factors.

As the ultimate goal of any adjuvant therapy is to decrease the chance of recurrence by eradicating hidden malignant cells after surgery, a longer interval between surgery and adjuvant CT might facilitate the proliferation of micrometastases. There is also a strong biological rationale for the early activation of CT after curative surgery. Studies in animal models have shown that removal of the primary tumor can increase the number of circulating tumor cells and accelerate the growth of residual cells [44]. Additionally, surgery has been shown to enhance the production of oncogenic growth factors (i.e. transforming growth factor α) and to significantly reduce the immunotherapeutic effects of interleukin-2 and lymphokine-activated killer cells [45,46]. 

The kinetics of cellular proliferation also indicate that in vivo, the growth rate is at first rapid and then slows progressively [47]. Therefore, at least theoretically, early cytotoxic treatment is expected to be beneficial. Finally, according to the historic mathematic model by Goldie and Coldman [48], drug sensitivity is related to mutation rate and as tumor mutation rate increases over time, a longer time interval after surgery might increase the probability of the appearance of a resistant phenotype. For all these reasons, CT will be more effective if initiated promptly when tumor burden is low.

Unfortunately, in gastrointestinal malignancies, adjuvant therapy is often delayed due to post-surgical complications and poor general conditions. In particular, patients undergoing gastric and pancreatic surgery frequently present significant nutritional problems that compromise their adequate recovery and the subsequent initiation of adjuvant CT. In this regard, minimally invasive surgical techniques, such as laparoscopic gastrectomy, may help by accelerating recovery and facilitating a prompt return to normal bowel function and an early discharge from hospital [49]. Consequently, patients will have easier access to potentially curative adjuvant treatments.

As for colorectal and gastric cancer, the results of this meta-analysis are consistent with previous reports showing that eight weeks after surgery is a reasonable cut-off for recommending adjuvant CT activation. Specifically, a meta-analysis of 10 published studies involving 15,410 patients concluded that delaying adjuvant CT beyond 12 weeks after surgery was associated with decreased survival among patients with resected colorectal cancer [40]. Similarly, a recent study conducted in Asia involving 840 D2-resected stage 2 and 3 gastric cancer patients showed that delayed treatment of adjuvant CT after eight weeks was associated with worse survival outcomes than early and intermediate treatment initiation. Therefore, the start of adjuvant CT should be considered within eight weeks after radical resection [29].

A pancreatectomy is a surgical procedure associated with high rates of complications that negatively affect both time to adjuvant treatment initiation and long-term outcomes. A post-hoc analysis of the largest trial of adjuvant CT for pancreatic cancer (i.e., ESPAC-3) did not show any overall survival difference for patients who started CT earlier than eight weeks (compared to 12 weeks) following surgery [36]. The only prognostic factor was represented by the completion of all six cycles of planned adjuvant CT. Three subsequent large retrospective studies were concordant in demonstrating no detriment to survival for patients who had delayed initiation of adjuvant therapy greater than 12 weeks after surgery [50,51]. Consistent with these findings, our meta-analysis did not show any significant survival benefit associated with early initiation, confirming that in pancreatic cancer, factors related to the biology of the tumor may play a major role. 

Our results need to be interpreted in the context of the study’s strengths and limitations. As a randomized controlled trial comparing the effect on survival of two different timings of initiation of adjuvant CT after surgery would not be feasible for ethical and clinical reasons, most of the included studies are derived from retrospective observations. Second, the reason for delaying CT for more than 6–8 weeks is unknown and could be potentially related to slow recovery after surgery or other morbidities. Eventually, the delay may potentially weaken the benefit of adjuvant therapy. Finally, the bad prognosis and the limited utility of older CT schedules can explain the negative effect of early initiation in pancreatic cancer. Otherwise, we can confirm that delaying adjuvant CT is potentially detrimental in stage III colorectal cancer and that this paper updates previous meta-analyses related to this topic with large case series. Furthermore, we can also provide evidence that in gastric cancer, if neoadjuvant CT is not scheduled, earlier initiation of postoperative CT could be useful. 

## 4. Materials and Methods

### 4.1. Search Strategy and Inclusion Criteria

The current systematic review was conducted in accordance with the Preferred Reporting Items for Systematic Review and Meta-Analyses statement [52]. We searched for relevant studies through database queries in PubMed, EMBASE, and the Cochrane Library (from inception to 16 December 2018) using the Medical Subject Headings: *adjuvant chemotherapy AND (colorectal neoplasms or stomach neoplasm or pancreatic neoplasm) and survival and (timing OR initiation OR delay OR start OR time OR interval).* Literature references were also scanned manually. To be eligible, studies had to include patients with resected gastric, colorectal, and pancreatic cancer and to assess the relationship between shorter (<6–8 weeks) and longer (>6–8 weeks) elapsed periods from surgery to the start of adjuvant CT and OS. Studies were excluded if they were abstracts or case reports, if they included patients treated with neoadjuvant therapy, and if they were written in a non-English language. 

### 4.2. Data Extraction

**Figure 5:**
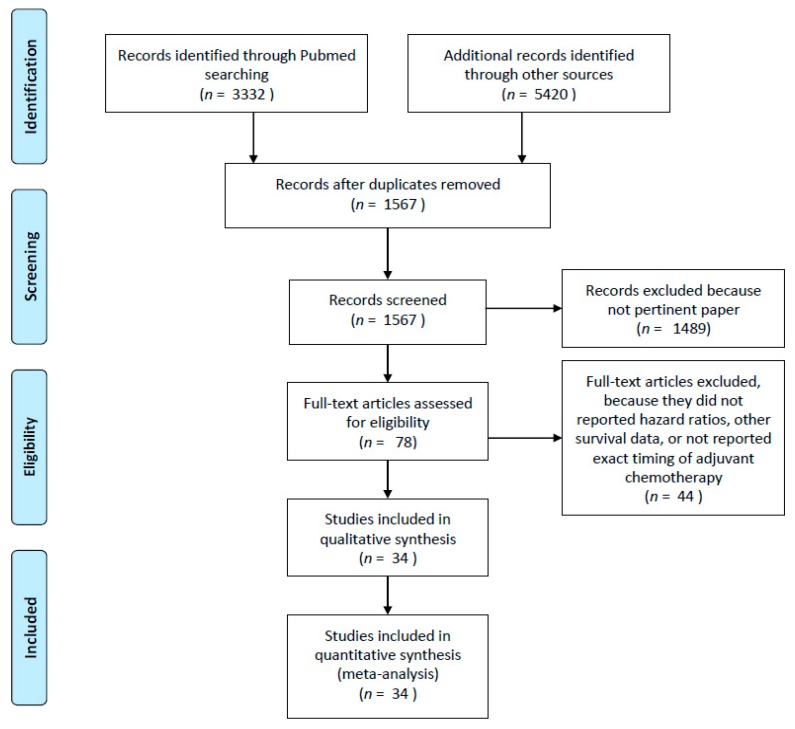
Flow diagram of included studies.

Based on the title, keywords, and abstract, three reviewers (F.P., A.G., and M.G.) selected the studies by applying the inclusion criteria. When there was doubt regarding whether or not to select a study, a discussed was conducted to resolve this. The three reviewers assessed the full versions of the selected articles. When disagreements about inclusion were not resolved by consensus, a senior reviewer (A.Z.) was consulted. Figure 5 outlines the identification of studies for this systematic review and meta-analysis. 

The three reviewers extracted data from the included studies. For each article, the following data were extracted using Microsoft Word spreadsheets: Author and year of publication; the number of participants; country; stage of disease; description of the comparison; and outcome measures available. A quality assessment of all the studies included in the meta-analysis was performed according to the Newcastle–Ottawa scale (NOS). The total scores ranged from 0 (worst) to 9 (best) for cohort studies, with a score of at least 7 indicating high quality.

### 4.3. Statistical Analysis

The measure of effect in all studies was the HR for OS. For each study, the HR and 95% CI was estimated depending on the data provided in the publication. When different intervals between surgery and the start of CT were presented, any event occurring in general beyond 6–8 weeks was compared with all events occurring within the 6–8 weeks interval. Three different forest plots were created for each disease (colorectal, gastric, and pancreatic cancer). The homogeneity assumption in the meta-analysis was assessed by the Cochrane Chi^2^ statistic and *I*^2^ statistics were calculated for each result. The pooled HRs for death with early vs delayed CT were calculated using the fixed effect model/Mantel–Haenszel (M-H) method when there was minimal heterogeneity in the variables among the studies, and the Der Simonian–Laird method (random effect model) when there was significant heterogeneity (*p* for heterogeneity <0.1).

Each publication was weighted as a function of the inverse variance of each effect size and Chi^2^ and *I*^2^ test methods were utilized for the between-study heterogeneity of the HRs. The statistically significant differences were defined as <0.1 for the Chi^2^ *p* and greater than 50% for the *I*^2^ test. The publication bias was evaluated through Egger’s linear regression, Begg’s rank correlation, and funnel plots and a *p*-value <0.05 for the Egger’s or Begg’s tests was considered representative of significant statistical publication bias. In addition, the trim and fill approach was used to obtain an adjusted effect size that took into account the publication bias. All statistical analyses were performed with Review Manager (RevMan) (computer program) Version 5.3 (Copenhagen: The Nordic Cochrane Centre, The Cochrane Collaboration, 2014).

## 5. Conclusions

Findings from our study demonstrate that starting adjuvant CT within 6–8 weeks post-surgery is associated with a significant survival benefit in colorectal and gastric cancer. These results suggest that the timing of CT initiation is an important variable and that great efforts should be made to minimize post-surgical recovery time.

## Figures and Tables

**Table 1:** Characteristics of included studies for colorectal cancer.

Author/ Year	*N*° pts	Type of Study	Median Follow up (months)	Country	Stage (%)	Comparison (weeks)	Type of Analysis	NOS Scale
Ahmed/ 2010 [4]	663	Retro	54.6	Canada	II–III (100)	< vs > 8	Multi	8
Bayraktar/ 2011 [5]	186	Retro	42.9	US	II–III (100)	< vs > 8	Multi	8
Becerra/ 2017 [6]	1133	Retro	NR	US	III (100)	< vs > 8	Multi	6
Berglund/ 2008 [13]	213	Phase III	NR	Sweden	III (100)	< vs > 8	Multi	NA
Bos/2015 [7]	6620	Retro	60	Netherlands	III (100)	< vs > 8	Multi	8
Chau/2005 [14]	801	Phase III	63.6	UK	II–III (100)	< vs 8–12	Multi	NA
Czaykowski/ 2011 [9]	345	Retro	69.8	Canada	III (100)	< vs > 8	Multi	8
Cheung/ 2009 [8]	6059	Retro	NR	Canada	II–III (100)	< vs > 8	Uni	6
Day/2014 [10]	209	Retro	30	UK	I–II (33), III (67)	< vs > 8	Multi	7
Dos Santos/ 2013 [11]	1318	Retro	41	Brazil	II–III (100)	< vs > 8	Multi	8
Gao/2018 [15]	9722	Retro	NR	US	III (100)	5–8 vs > 8	Multi	6
Hershman/ 2006 [16]	4382	Retro	NR	US	III (100)	< vs > 2–3 months	Multi	6
Kang/2013 [17]	159	Retro	41.5	Korea	III (100)	< vs 5–6	Multi	7
Kim/2017 [18]	5355	Retro	42.2	Korea	II–III (100)	< vs > 8	Multi	7
Klein/2015 [19]	1827	Retro	NR	Denmark	III (100)	4–8 vs > 8	Multi	6
Lima/2011 [20]	1053	Retro	NR	Canada	III (100)	< vs > 8	Multi	6
Massarweh/ 2015 [12]	51,331	Retro	NR	US	III (100)	8 vs 8–16	Multi	6
Nachiappan/ 2015 [21]	30,836	Retro	1–184*	UK	NR	< vs 8–16	Multi	7
Peixoto/ 2015 [22]	635	Retro	57.9	Canada	III (100)	< vs > 8	Multi	8
Sun/2016 [23]	7794	Retro	61	US	II–III (100)	< vs > 44 days	Multi	8
Tsai/2013 [24]	1054	Retro	72.5	Taiwan	III (100)	< vs ≥ 6	Multi	8
Zeig-Owens/ 2009 [25]	3006	Retro	≥48	US	II–III (100)	< vs > 45 days	Multi	8

* range of follow up; CSS, cancer-specific survival; Multi, multivariate; NOS, Newcastle–Ottawa scale; pts, patients; Retro, retrospective; RFS, relapse-free survival; Uni, univariate; vs, versus.

**Table 2:** Characteristics of included studies for gastric cancer.

Author/Year	*N*° pts	Type of Study	Median Follow up (months)	Country	Stage (%)	Comparison (weeks)	Type of Analysis	NOS Scale
Di Bartolomeo/ 2016 [26]	1072	Retro	56.9	Italy	Ib (8.2); II (31.8) III (41.6); IV (18.4)	< vs > 8	Multi	8
Fujitani/ 2017 [27]	498	Retro	NR	Japan	II (36.1); III (63.9)	< vs > 6	Multi	6
Greenleaf/ 2016 [28]	2332	Retro	NR	US	I (11); II (30); III (50)	8 vs > 8	Multi	6
Park/ 2015 [29]	840	Retro	34	Korea	II (28.6); III (71.4)	4–8 vs 8	Multi	7
Qu/ 2015 [30]	266	Retro	28	China	IB (4.1); II (28.2) III (67.7)	< vs > 6.4	Multi	6
Yamamoto/ 2016 [31]	113	Retro	47.6	Japan	II (34.5); III (65.5)	< vs > 6	Multi	7

Multi, multivariate; NOS, Newcastle–Ottawa scale; pts, patients; Retro, retrospective; vs, versus.

**Table 3:** Characteristics of included studies for pancreatic cancer.

Author/Year	*N*° pts	Type of Study	Median Follow up (months)	Country	Stage (%)	Comparison (weeks)	Type of Analysis	NOS Scale
Kim/2017 [32]	113	Retro	20.3	Korea	-	< 6 vs > 6	Multi	6
Lee/2017 [33]	311	Retro	28	Korea	I (4.1); II 94.2; III 1.6	< 6 vs > 6	Multi	7
Patel/2015 [34]	34	Retro	22	US	N0 38; N+ 62	< 8 vs > 12	Uni	6
Saeed/2016 [35]	420	Retro	19.3	US	I (8.5); II (87.1); III 4.2	< 8 vs > 8	Multi	7
Valle/2014 [36]	985	Phase III	58.7	Europe	I (10); II 29; III 58; IVa 4	< 8 vs > 8	Multi	8
Yabusaki/2016 [37]	168	Prosp	24.5	Japan	I–III (100)	< 8 vs > 8	Multi	7

Multi, multivariate; NOS, Newcastle–Ottawa scale; Prosp, prospective, pts, patients; Retro, retrospective; Uni, univariate; vs, versus.
